# Molecular Evolution and Increasing Macrolide Resistance of *Bordetella pertussis*, Shanghai, China, 2016–2022

**DOI:** 10.3201/eid3001.221588

**Published:** 2024-01

**Authors:** Pan Fu, Jinlan Zhou, Chao Yang, Yaxier Nijiati, Lijun Zhou, Gangfen Yan, Guoping Lu, Xiaowen Zhai, Chuanqing Wang

**Affiliations:** National Children's Medical Center, Shanghai, China (P. Fu, J. Zhou, Y. Nijiati, L. Zhou, G. Yan, G. Lu, X. Zhai, C. Wang);; Chinese Academy of Sciences, Shanghai (C. Yang)

**Keywords:** Bordetella pertussis, macrolide, antimicrobial resistance, vaccine-preventable diseases, genotype, Shanghai, China, bacteria

## Abstract

Resurgence and spread of macrolide-resistant *Bordetella pertussis* (MRBP) threaten global public health. We collected 283 *B. pertussis* isolates during 2016–2022 in Shanghai, China, and conducted 23S rRNA gene A2047G mutation detection, multilocus variable-number tandem-repeat analysis, and virulence genotyping analysis. We performed whole-genome sequencing on representative strains. We detected pertussis primarily in infants (0–1 years of age) before 2020 and older children (>5–10 years of age) after 2020. The major genotypes were *ptxP1/prn1/fhaB3/ptxA1/ptxC1/fim2–1/fim3–1* (48.7%) and *ptxP3/prn2/fhaB1/ptxA1/ptxC2/fim2-1/fim3-1* (47.7%). MRBP increased remarkably from 2016 (36.4%) to 2022 (97.2%). All MRBPs before 2020 harbored *ptxP1*, and 51.4% belonged to multilocus variable-number tandem-repeat analysis type (MT) 195, whereas *ptxP3*-MRBP increased from 0% before 2020 to 66.7% after 2020, and all belonged to MT28. MT28 *ptxP3*-MRBP emerged only after 2020 and replaced the resident MT195 *ptxP1*-MRBP, revealing that 2020 was a watershed in the transformation of MRBP.

Whooping cough (pertussis) is a contagious respiratory illness caused by *Bordetella pertussis*. The introduction of the whole-cell vaccine (WCV) successfully decreased the incidence of pertussis. Although vaccination has been successful, replacement of the WCV with an acellular vaccine (ACV) has correlated with reemergence of pertussis, especially in adolescents and infants (*1*). In China, ACV was developed in the late 1990s and has replaced WCV and been exclusively used in China since 2012 (*2*,*3*). However, a multicenter study showed that the levels of protective antibodies against pertussis were already very low in immunized children 2–20 years of age (*4*).

Resurgence of pertussis has been widely reported and is mainly found in age groups of unvaccinated or incompletely vaccinated children or those whose immunity has waned (*5*,*6*). Mooi et al. (*7*,*8*) first identified the antigenic divergence between circulating isolates and vaccine strains in 1998, which explained the reemergence of pertussis and the distinct epidemiology of pertussis in different regions. Since then, a series of studies have demonstrated antigenic changes in bacterial virulence genes that might compromise vaccine-mediated immunity against *B. pertussis* (*9*–*11*). Virulence antigens, such as filamentous hemagglutinin (FHA), pertactin (Prn), pertussis toxin (PT), fimbriae2 (Fim2), and fimbriae3 (Fim3), are the essential components of ACV (*12*). PT export genes are regulated by the *ptx* promoter (*ptxP*) and may be required for efficient translation of *ptx* mRNA in *B. pertussis* strains (*13*). The *ptxP* region include 2 major alleles *ptxP1* and *ptxP3*, and *ptxP3* produces more PT than the *ptxP1* allele (*14*).

In many countries, circulating *B. pertussis* harbors different virulence genotypes compared with vaccine strains (*15*,*16*). Different alleles of *ptxP*, *fhaB, ptxA, ptxC*, *fim2*, and *fim3* have been reported in many studies (*6*,*17*,*18*). Among those virulence-related genes, the *ptxC* alleles *ptxC1* and ptxC2 have been described; those alleles differ at a single nucleotide, resulting in a silent mutation (*19*). Compared with the major *ptxP1*/*fhaB1/prn1*/*ptxA2* genotype of vaccine strains, the *ptxP3*/*fhaB3*/*prn2*/*ptxA1* genotype have emerged in the circulating *B. pertussis* population in China (*6*,*20*,*21*).

Despite the variation in virulence genotypes in circulating strains, different *B. pertussis* subtypes are prevalent in the world. The multilocus variable-number tandem-repeat analysis (MLVA) type (MT) 27 strain carrying the genotype of *ptxP3/ptxA1/prn2/fm3–1* has become the predominant *B. pertussis* strain in many countries (*22*). However, MT27 has seldomly been reported in China, whereas the MT55, MT195, or MT104 strains harboring the *ptxP1* allele have been reported to circulate in some regions of China (*23*,*24*). Macrolide-resistant *B. pertussis* (MRBP), which carries an A-to-G transition at nucleotide position 2047 (A2047G mutation) in a region critical for erythromycin binding, emerged in some countries, but was only frequently detected in China (*15*,*16*,*25*–*27*). MRBPs generally carry *ptxP1* and *fhaB3*, but 2 novel MRBPs belonging to MT28 and MT27 carrying *ptxP3* and *fhaB1* were reported in mainland China (*15*,*28*).

Our recent study reported that MT28 *ptxP3*-MRBP has emerged and spread in Shanghai, China, during 2021–2022 (*29*). However, several urgent questions remain to be resolved. For example, was *ptxP3*-MT28 MRBP dominant in Shanghai in the long term, or did it emerge in 2021 and 2022? Why and when did *ptxP3*-MT28 MRBP emerge in Shanghai, and how did they evolve? To resolve those questions, we conducted further research during 2016–2022 to reveal the evolution of MRBP in Shanghai. We collected a total of 283 *B. pertussis* isolates during 2016–2022 in Shanghai and systematically analyzed the antimicrobial resistance and molecular evolution of those strains. 

## Methods

### Enrollment of Case-Patients with *B. pertussis* Infection

We included in the study a total of 1,065 children admitted to the Children’s Hospital of Fudan University and diagnosed with pertussis during January 2016–October 2022, who had nasopharyngeal swab (NP) samples collected and delivered to the microbiology laboratory for *B. pertussis* culture, antimicrobial resistance testing, and PCR detection. We extracted DNA from NP samples and performed real-time PCR (LightCycler 480; Roche, https://www.roche.com) to detect nucleic acids according to the protocol of a pertussis bacteria nucleic acid detection kit based on the PCR-fluorescent probe method (Yilifang Biotechnology, http://www.yilifangbio.com). The laboratory testing results and data collection were based on electronic medical records during hospitalization or clinic visits, and all data analysis was anonymous. The study protocol was approved by the Ethics Committee of the Children’s Hospital of Fudan University (approval no. 2022-66).

### PCR and Sequencing for 23S rRNA A2047G Mutation Detection and Virulence Genotyping Analysis

We obtained 692 *B. pertussis* strains in 2016 (11 strains), 2017 (177 strains), 2018 (165 strains), 2019 (169 strains), 2020 (1 strain), 2021 (30 strains), and 2022 (139 strains). Because very few strains were obtained from 2016, 2020, and 2021, we selected all 42 strains for this study. We chose other isolates by the systematic sampling method, yielding 50 strains in 2017, 45 strains in 2018, 74 strains in 2019, and 72 strains in 2022. We gave each strain a number and then chose it by a random method to ensure each strain had an equal chance of being chosen through the use of an unbiased selection method. We selected a total of 283 isolates for further analysis.

We prepared genomic DNA of *B. pertussis* isolates by using a QIAamp DNA Mini Kit (QIAGEN, https://www.qiagen.com). We performed PCR-based sequencing of the A2047G mutation as described in a previous study (*30*). We also performed PCR and sequencing of virulence-related genes (*ptxP*, *ptxA, ptxC*, *prn, fim2, fim3*) as previously described (*6*). By using a convention for *fhaB* allele naming that defined *fhaB1* and *fhaB2* alleles by the A2493C mutation and defined the novel *fhaB3* allele by the C5330T mutation, as previously described (*23*,*31*), we identified *fhaB* alleles by detecting and sequencing these 2 mutations. The primers for *fhaB-*2493 were forward, 5′-GATGTAGGCAAGGTTTCCGC-3′, and reverse, 5′-CGCTCGACACATGCAGAC-3′; the primers for *fhaB-*5330 were forward, 5′-ATATCGACAACAAGCAGGCC-3′, and reverse, 5′-TTGACATAGCCGATACCGCT-3′. We retrieved reported DNA sequences from GenBank and analyzed them by using BLAST (https://blast.ncbi.nlm.nih.gov) to determine the allele of each virulence gene.

### MLVA 

We performed MLVA by following the procedures described by Schouls et al. (*32*). We amplified 5 loci (variable-number tandem-repeat [VNTR] 1, VNTR3a/VNTR3b, VNTR4, VNTR5, and VNTR6) by using PCR detection. We calculated the number of repeats at each VNTR locus from the DNA fragment length. We assigned an MT on the basis of the combination of repeat counts for VNTRs 1, 3a, 3b, 4, 5, and 6, as described in previous reports (*15*,*32*).

### DNA Extraction and Whole-Genome Sequencing

We further subjected 4 representative BP strains, including 1 MT27 *ptxP3* macrolide-sensitive *B. pertussis* (MSBP) (BP1-Shanghai-2016), 1 MT195 *ptxP1*-MRBP (BP7-Shanghai-2016), 1 MT28 *ptxP3*-MSBP (P20-Shanghai-2017), and 1 MT28 *ptxP3*-MRBP (P745-Shanghai-2022) to whole-genome sequencing (WGS) analysis. We extracted genomic DNA by using the sodium dodecyl sulphate method (*33*). We constructed libraries for single-molecule real-time sequencing with an insert size of 10 kb by using the SMRTbell Template Prep Kit 1.0 (PacBio, https://www.pacb.com). We generated sequencing libraries for the Illumina platform by using the NEBNext Ultra DNA Library Prep Kit for Illumina (New England BioLabs, https://www.neb.com). We sequenced the whole genomes by using the PacBio Sequel platform and Illumina NovaSeq PE150 at Beijing Novogene Bioinformatics Technology Co., Ltd (Beijing, China). We deposited the sequencing data into GenBank (accession nos. CP118023–6).

### Public Genome Dataset

We included a total of 1,491 public genomes of *B. pertussis* strains from China (*15*,*21*,*28*) and global *B. pertussis* P strains in this study for comparison (Appendix 1 Table). We sequenced the public genomes for various purposes, and they covered 27 countries from 8 geographic areas (Appendix 1 Table). We downloaded raw short-read sequencing data from the National Center for Biotechnology Information Sequence Read Archive (https://www.ncbi.nlm.nih.gov/sra). We filtered sequencing reads by using Trimmomatic (*34*), and we performed de novo genome assembly of public data by using SPAdes (*35*) with default settings.

### Single-Nucleotide Polymorphism Calling and Phylogenetic Analysis

We identified core-genome (regions present in >99% of isolates) single-nucleotide polymorphisms (SNPs), as previously described (*36*). In brief, we aligned the assemblies against the reference genome (GenBank accession no. NC_002929.2, Tohama I) by using MUMmer (*37*) to generate whole-genome alignment. We performed SNP calling by using SNP-Sites (*38*) on the basis of the alignment. We identified the repetitive regions of the reference genome by using Tandem repeats finder and self-aligning by blastn (https://blast.ncbi.nlm.nih.gov). We excluded SNPs located in repetitive regions from further analysis. We constructed a maximum-likelihood phylogenetic tree by using RAxML-NG (*39*) under the general time-reversible with gamma distribution model.

### Statistical Analysis

We analyzed data by using the t test, χ^2^ test, or Fisher exact test, as appropriate. We performed all statistical analyses by using the SPSS Statistics 13.0 (IBM, https://www.ibm.com). We considered p<0.05 to be statistically significant.

## Results

### Clinical Characteristics of Children with BP Infection

**Figure 1:**
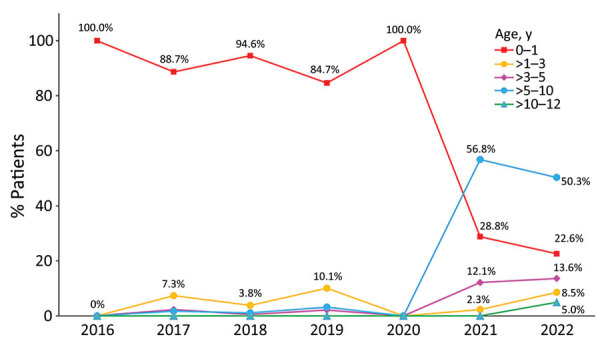
Distributions of pertussis patients in different age groups, Shanghai, China, 2016–2022. Pertussis was primarily detected from infants (0–1 years of age) before 2020 but mostly from older children and adolescents (>5–10 years of age) after 2020.

A total of 1,065 children had pertussis diagnosed at the Children’s Hospital of Fudan University during January 2016–October 2022 (Appendix 2 Table 1). Of those, 65.0% (692) had culture-proven pertussis, and the others were culture-negative but verified by PCR or clinical symptoms. The case-patients were 470 girls (44.1%) and 595 boys (55.9%), and the average age was 2.6 years (range 23 days–11.5 years). Approximately 93.8% of the case-patients (999) had cough symptoms; average duration of cough was of 20.7 days (range 1–130 days). Most of the patients (75.5% [804]) were treated with antibiotics before sampling, among which macrolides were used in 60.1% (640) of patients. The age distributions of pertussis changed from 2016 to 2022; pertussis was detected primarily in infants (0–1 years of age) (84.7%–100%) before 2020 but was mostly detected in older children and adolescents (>5–10 years of age) (50.3%–56.8%) after 2020 (p<0.001) (Figure 1). 

### MLVA Types of *B. pertussis* strains

We identified 14 MLVA types in this study, and the major MLVA types were MT195 (26.9%), MT28 (26.1%), MT27 (20.8%), MT104 (13.4%), and MT55 (6.4%) (Appendix 2 Table 2). The other MLVA types were MT158 (1.1%), MT 16 (1.1%), MT29 (0.7%), MT114 (0.7%), MT30 (0.4%), MT32 (0.4%), untyped-1 (0.4%), untyped-2 (1.1%), and untyped-3 (0.4%). 

**Figure 2:**
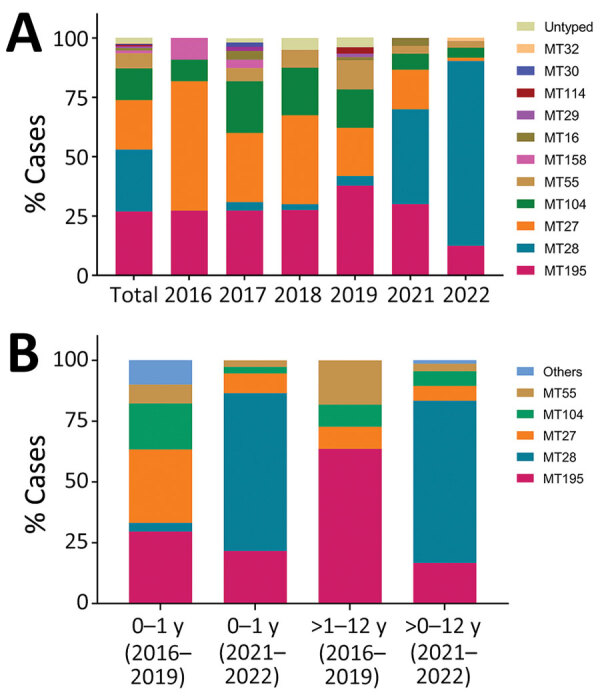
Distributions of prevalent *Bordetella pertussis* subtypes over time (A) and by age group (B), Shanghai, China, 2016–2022. Fourteen MTs were identified in this study. MT27 was the major strain during 2016–2019, whereas MT28 isolates increased quickly during 2021–2022 (panel A). MT distributions in infants (0–1 years of age) and noninfants (>1–12 years of age) change substantially from 2016–2019 to 2021–2022 (panel B). MT, multilocus variable-number tandem-repeat analysis type.

Only 1 strain was isolated in 2020, so we deleted the analysis of 2020. MT27 was the main subtype during 2016–2019 (29.1%–54.5%). However, MT28, which accounted for 0–4.1% before 2020, accounted for 40.0% in 2021 and 77.8% in 2022 (Figure 2, panel A).

We further analyzed the MT distributions in different age groups. MT28, which was seldomly detected in infants (3.6% [6/169]) and absent in noninfants (>1–12 years of age) (0% [0/11]) during 2016–2019, was predominantly isolated from all age groups during 2021–2022, accounting for 64.9% (24/37) in infants, and 66.7% (44/66) in noninfants (Figure 2, panel B).

### Virulence Gene Alleles and Genotype Profiles of *B. pertussis* Strains

We identified 2 *ptxP* alleles; we identified *ptxP1* in 49.8% and *ptxP3* in 50.2% of *B. pertussis* strains. *ptxP3*, which accounted for only 37.2% before 2020, became the major allele (73.5%) after 2020. Moreover, we identified 4 types of *prn* (*prn1*, *prn2*, *prn3*, and *prn9*), 2 types of *fhaB* (*fhaB1* and *fhaB3*), 3 types of *ptxC* (*ptxC1*, *ptxC2*, and *ptxC3*), 1 type of *ptxA* (*ptxA1*), 1 type of *fim2* (*fim2–1*), and 3 types of *fim3* (*fim3–1*, *fim3–2*, and *fim3–4*).

*ptxP1* was mostly linked to *prn1* and *fhaB3*, whereas *ptxP3* linked closely to *prn2* and *fhaB1.* The major genotypes were *ptxP1/prn1/fhaB3/ptxC1/ptxA1/fim2–1/fim3–1* (48.7%) and *ptxP3/prn2/fhaB1/ptxC2/ptxA1/fim2–1/fim3–1* (47.7%): the former included 7 subtypes (MT16, MT27, MT30, MT55, MT104, MT195, and untyped-3), and the latter involved 6 subtypes (MT27, MT28, MT32, MT114, MT158, and untyped-2). (Appendix 2 Table 3).

### A2047G mutation and Antimicrobial-Resistance Profiles of *B. pertussis* Strains

*B. pertussis* was highly resistant to macrolides, and MRBP accounted for 72.4% (205/283) of strains (Table). A total of 97.2% of *ptxP1/prn1/fhaB3*-BP and 91.9% of MT28 *ptxP3/prn2/fhaB1*-BP belonged to MRBP, whereas all non-MT28 *ptxP3/prn2/fhaB1*-BP were MSBP.

**Table:** Antimicrobial-resistance profiles and virulence genotypes of 283 *Bordetella pertussis* isolates, Shanghai, China, 2016–2022*

Antibiotic	MIC, μg/mL	Total, no (%)	Frequency of genotype profiles, no (%)		
			*ptxP1/prn1/fhaB3* non-MT28, n = 141	*ptxP3/prn2/fhaB1* non-MT28,† n = 68	*ptxP3/prn2/fhaB1* MT28,‡ n = 74
Erythromycin	Resistant, >256	205 (72.4)	137 (97.2)	0	68 (91.9)
	Sensitive, <0.064	78 (27.6)	4 (2.8)	68 (100)	6 (8.1)
Azithromycin	Resistant, 128 to >256	205 (72.4)	137 (97.2)	0	68 (91.9)
	Sensitive, <0.064	78 (27.6)	4 (2.8)	68 (100)	6 (8.1)
Clarithromycin	Resistant, 128 to >256	205 (72.4)	137 (97.2)	0	68 (91.9)
	Sensitive, <0.064	78 (27.6)	4 (2.8)	68 (100)	6 (8.1)
Sulfamethoxazole/ trimethoprim	Resistant, >32	0	0	0	0
	Sensitive, 0.064 to <0.008	283 (100)	141 (100)	68 (100)	74 (100)

*MT, multilocus variable-number tandem-repeat analysis type. †Includes 1 MT27-*ptxP3/prn3/ptxC2* strain. ‡Includes 1 MT28*-ptxP3/prn9/fhaB1* strain.

We frequently detected the A2047G mutation in 205 *B. pertussis* strains (72.4%) that showed 100% resistance to erythromycin, azithromycin, and clarithromycin. The A2047G mutation accounted for 61.0% before 2020 and 93.1% after 2020. All MT195, MT55, and MT104 carried the A2047G mutation, but none of the MT27 acquired this mutation. A2047G mutation in MT28 increased from 0% before 2020 to 100% after 2020.

**Figure 3:**
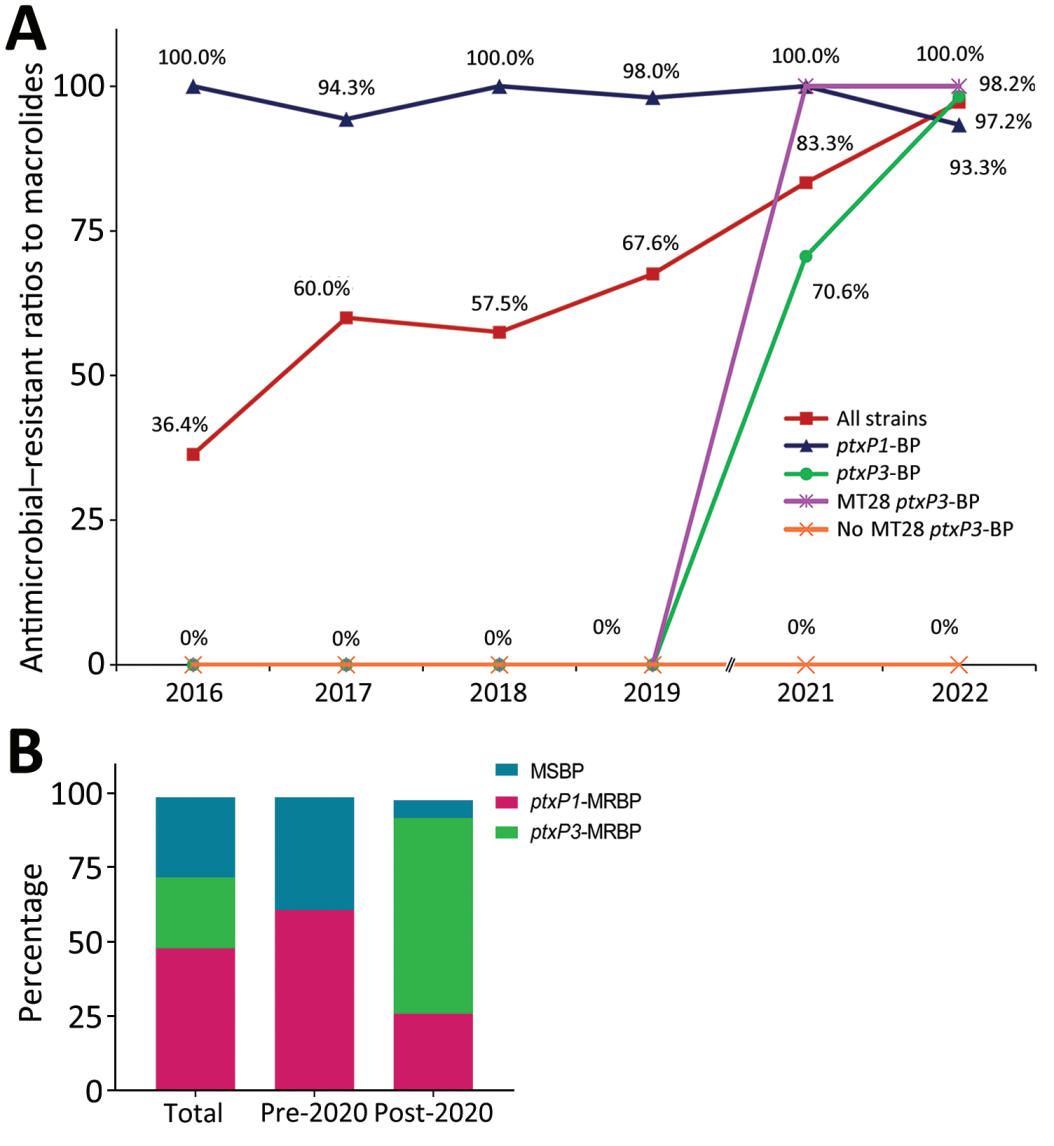
Changing macrolide resistance of circulating *Bordetella pertussis* strains, Shanghai, China, 2016–2022. A) *ptxP3*-strains showed very high resistance to macrolides after 2020. Resistance to macrolides was different in non-MT28 (0%) and MT28 (100%) isolates. B) Percentages of macrolide-sensitive BP, *ptxP1*-MRBP, and *ptxP3*-MRBP before and after 2020 show that *ptxP1*-MRBP strain was prevalent before 2020 but predominately *ptxP3-*MRBP spread after 2020. MRBP, macrolide-resistant *Bordetella pertussis*; MT, multilocus variable-number tandem-repeat analysis type.

**Figure 4:**
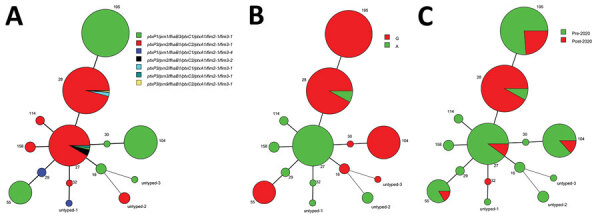
Minimum-spanning tree of 283 *Bordetella pertussis* MTs, Shanghai, China, 2016–2022. A) Virulence genotype profiles; B) A2047G mutations; C) pre-2020 versus post-2020. Circle sizes indicate the number of each MT. Differences in the length and thickness of the lines linking 2 circles indicate differences in the number of variable-number tandem repeats between the 2 linked MTs. MT, multilocus variable-number tandem repeat analysis type.

MRBP increased from 36.4% in 2016 to 97.2% in 2022, including *ptxP1*-MRBP (48.4% [137/283]) and *ptxP3*-MRBP (24.0% [68/283]). Most (100% in 2016, 2018, and 2021; 94.3% in 2017; 98.0% in 2019; and 93.3% in 2022) of the *ptxP1 *strains belonged to MRBP. However, macrolide resistance in *ptxP3* strains increased from 0% before 2020 to 70.6% in 2021 and 98.2% in 2022. Of note, macrolides resistance in MT28 *ptxP3-*strains switched from 0% before 2020 to 100% after 2020, whereas all non-MT28 *ptxP3* isolates showed sensitivity to macrolides (Figure 3, panel A). *ptxP1*-MRBP was prevalent before 2020 (111 [61.7%]); of those 111 strains, of which 57 (51.4%) were MT195. *ptxP3*-MRBP, which was absent before 2020, increased to 66.7% after 2020, and all of them belonged to MT28 (Figure 3, panel B; Figure 4).

### Combination of MLVA Types, Virulence Genotypes, and A2047G Mutations

MT195, MT55, and MT104 all carried *ptxP1/prn1/fhaB3* and the A2047 mutation (Figure 4). As 2 closely related MLVA types, 98.3% of MT27-BP and 98.6% of MT28-BP carried the genotype of *ptxP3/prn2/fhaB1*. However, the A2047G mutation was highly detected in MT28 (91.9%) but absent (0%) in MT27 (Figure 4, panels A, B). MT195, MT27, and MT104 were the major subtypes before 2020, whereas MT28 emerged and spread quickly after 2020 (Figure 4, panel C).

### WGS Analysis

**Figure 5:**
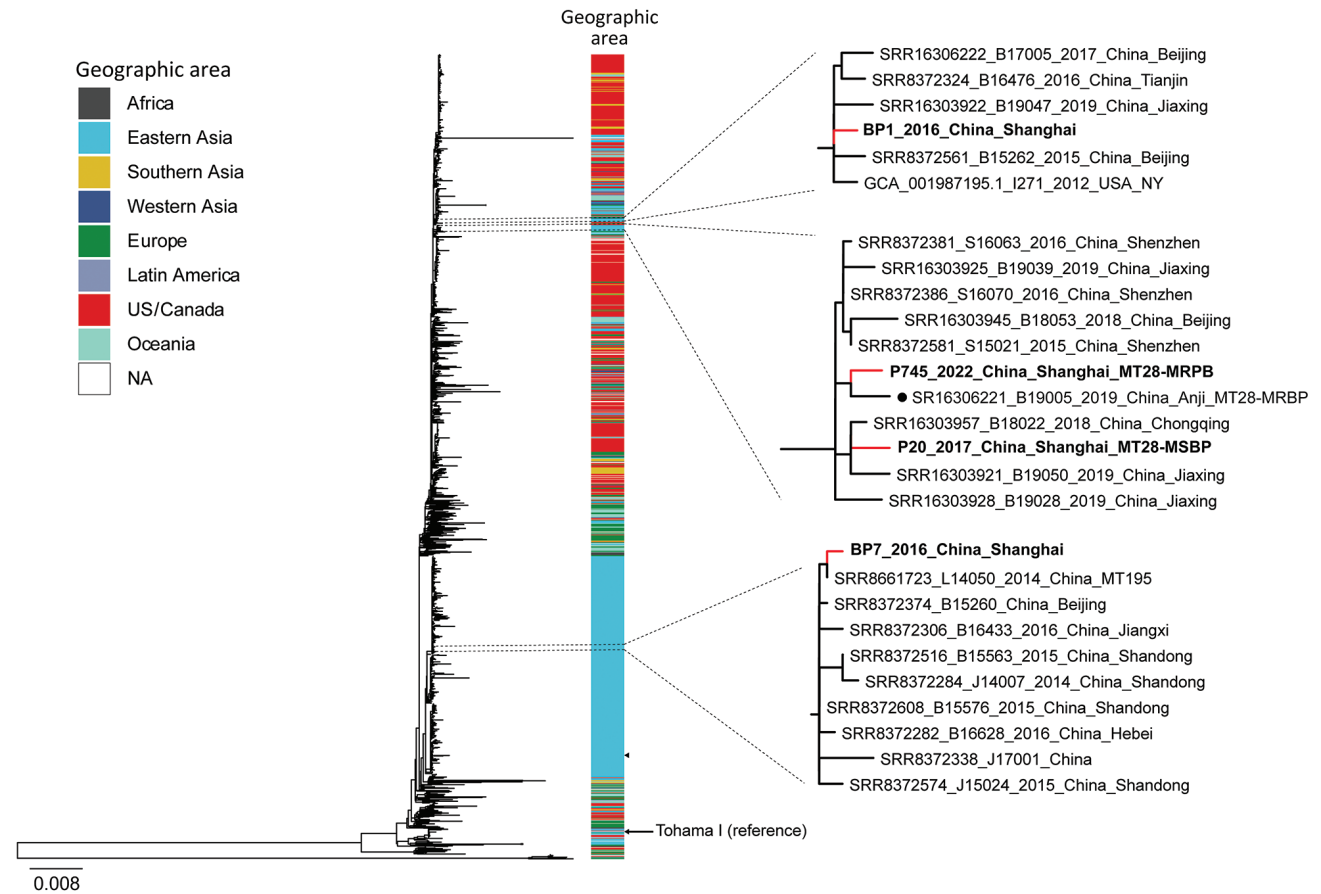
Maximum-likelihood phylogenetic tree of 4 Shanghai, China, and 1,491 global *Bordetella pertussis* strains, by geographic area, 2016–2022. Red lines indicate Shanghai strains; bold indicates 2 MT28 strains. Black dot indicates MT28-MRBP (B19005) strain from Anhui Province, China. Arrow indicates reference strain Tohama I. Shanghai strains associated phylogenetic subclades are enlarged for visualization. MSBP, macrolide-sensitive *Bordetella pertussis*; MT, multilocus variable-number tandem-repeat analysis type; NA, not applicable.

Four *B. pertussis* strains (MT27 *ptxP3*-MSBP, MT195 *ptxP1*-MRBP, MT28 *ptxP3*-MSBP, and MT28 *ptxP3*-MRBP) were chosen for further WGS analysis. We constructed a maximum-likelihood phylogenetic tree of 4 Shanghai and 1,491 global strains. *B. pertussis* isolates in Shanghai were closely related to other isolates from China but differed from other international strains isolated from the United States, Europe, Australia, Argentina, Africa, Japan, Iran, Israel, and other regions and countries (Figure 5). Of note, MT28-MRBP (P745) was prevalent after 2020 and was closely related to MT28 *ptxP3*-MSBP (P20) but was quite heterogeneous to MT195-MRBP (BP7) and MT27 *ptxP3*-MSBP (BP1) before 2020. Moreover, P745 was highly homologous to a previously reported MT28-MRBP (B19005) in Anhui Province, China.

## Discussion

In this study, we systematically investigated the clinical characteristics, antimicrobial resistance profiles, and molecular evolution of *B. pertussis* strains in Shanghai, China, during 2016–2022. Pertussis was primarily diagnosed in infants before 2020 but mostly in older children and adolescents after 2020. MRBPs remarkably increased, from 36.4% in 2016 to 97.2% in 2022. MT28 *ptxP3/prn2/fhaB1*-MRBP emerged only after 2020 and replaced MT195 *ptxP1/prn1/fhaB3*-MRBP, which was prevalent before 2020, indicating that 2020 was a watershed in the transformation of MRBP in Shanghai, China.

The first MRBP in China was reported in Shandong Province in 2011 (*40*). MRBPs in China was thought less likely to cause epidemics in other countries because the MRBPs in China were mostly assigned to *ptxP1* lineage, whereas *ptxP3* strains are currently endemic in other countries (*21*,*27*). In China, *ptxP1*-MRBPs were reported to contribute 75.4% (Zhejiang Province, 2016), 48.6% (Shenzhen Province, 2015–2017), and 84.9% (a multicenter study during 2017–2019) of the circulating *B. pertussis* strains in China (*15*,*41*,*42*). Previous studies showed that MRBP was mostly linked to the *ptxP1* allele and that the *ptxP3* strain was isolated from MSBP without exception (*6*,*20*,*41*). Our recent study demonstrated that the *ptxP3* allele had a close linkage with MRBP (*29*). In this study, *ptxP1-*MRBP was the major (61.7%) strain during 2016–2020, whereas *ptxP3-*MRBP, which emerged only after 2020, replaced *ptxP1-*MRBP and became predominant (66.7%) after 2020.

MRBP strains were widely prevalent in western China and mainly linked to MT195, MT104, and MT55 (*26*). Wu et al. (*15*) showed that MT28 MRBP with genotype of *ptxP3/fhaB1/prn9* was first identified in Anhui Province, China, revealing the emergence of *ptxP3*-MRBP in mainland China (*15*). In this study, the circulating *B. pertussis* strains changed greatly from 2016 to 2022. MT195 presented the VNTR profiles of 8-6-0-7-6-8, whereas MT28 showed the profiles of 8-7-0-7-6-8, and MT27 showed the profiles of 8-7-0-7-6-7. Although those subtypes have minor differences on VNTR3a or VNTR6, their virulence genotypes and A2047G mutation carriages were quite different, making the circulating strains very heterologous. All MRBPs before 2020 harbored *ptxP1* and 51.4% belonged to MT195, whereas *ptxP3*-MRBP, which was absent before 2020, increased to 66.7% after 2020, and all belonged to MT28. WGS analysis further revealed that MT28-MRBP was quite heterologous with MT195-MRBP, revealing the different molecular characteristics of MRBP prevalent before and after 2020 in Shanghai.

MT28-MRBP in this study was quite different from the international strains but represented close relevance to MT28-MSBP isolated before 2020, which indicates that MT28-MRBP was not reported from other countries but more likely because the resident MT28-MSBP acquired the A2047G mutation and became resistant to macrolides. Moreover, the emergence and spread of MT28 *ptxP3*-MRBP in Shanghai were probably related to the selection pressure from high usage of macrolides and vaccination. Macrolides were excessively used for treating pertussis, which might participate in the selection of *ptxP3*-MRBP. Of interest, although MRBPs are highly resistant to macrolides, most (60.1%) of the MRBP patients were still treated with macrolides in this study. In addition, compared with vaccine strains in China with the genotype of *ptxP1/fhaB1/prn1/ptxA2/ptxC1*, MT28 harbored more gene variants, including *ptxP3*, *prn2*, *ptxA1*, and *ptxC2* than MT195, which carried *fhaB3* and *ptxA1.* Currently, 2 types of diphtheria, tetanus, and pertussis (DTaP) vaccine formulations are licensed in China: one is the 2-component DTaP vaccine containing PT and FHA, another is the 3-component DTaP vaccine containing PT, FHA, and PRN (*43*). The circulating *B. pertussis* has evolved, mainly changed from *ptxP1* to *ptxP3* lineage, indicating the *ptxP3* variation reflect selective advantage under high coverage with acellular pertussis vaccine (*42*). Previous study showed that *prn2* variation affected the efficacy of commercial vaccine, and mice studies suggested that the incorporation of *prn2* to vaccine could enhance the ACV’s efficacy (*44*). Moreover, studies from Safarchi et al. (*45*) and Van Gent et al. (*46*) demonstrate that *ptxP3/prn2*-BP colonized better than the *ptxP1/prn3*-BP strain and provide the evidence for increased fitness and better immune evasion of *ptxP3/prn2* strains in a mouse model involving mice immunized with 3-component ACVs. Therefore, we hypothesized that *prn2* and *ptxP3* variation in MT28 strains may play a role in better fitness and immune evasion compared with ACVs in China, causing MT28-BP to be selected by the vaccination and then to spread quickly. The exact relationship between *prn2*/*ptxP3* variant and vaccine escape needs further study.

In this study, pertussis was primarily detected in infants before 2020 but was mostly detected in older children after 2020. We propose 2 potential hypotheses for this age shift. First, the age shift was closely related to the emergence of MT28-MRBP; *ptxP3/ptxA1/ptxC2/prn2*-carrying MT28 strains, which emerged and spread after 2020 could avoid the immunity of vaccine and weaken the vaccine effects, making the pertussis populations shift from unvaccinated or incompletely vaccinated infants to vaccinated population. Second, the COVID-19 pandemic increased the public awareness of microbiologic laboratory testing in children with respiratory symptoms, so more older children who were not considered as the primary pertussis population before 2020 accepted *B. pertussis* testing and were diagnosed with pertussis after 2020, making 2020 become the watershed moment for the shift of pertussis population.

In conclusion, we systematically investigated the molecular evolution of MRBPs to clarify the evolution of MRBP from MT195 to MT28 in Shanghai, China, during 2016–2022, revealing that 2020 was watershed in the transformation of MRBPs from MT195 *ptxP1/prn1/fhaB3*-alleles to MT28 *ptxP3/prn2/fhaB1*-alleles in Shanghai. The emergence and spread of MT28 *ptxP3-*MRBP strains are likely attributable to the A2047G mutation and the selection pressure from vaccination and high usage of macrolides, which will further complicate the epidemiology of pertussis and evolve to pose a looming threat to global public health. Therefore, worldwide surveillance of the molecular evolution and AMR profiles of circulating *B. pertussis*, especially *ptxP3-*MRBP, is urgent.

Appendix 1 Genomes used in study of molecular evolution and increasing macrolide resistance of *Bordetella pertussis*, Shanghai, China, 2016–2022.

Appendix 2 Additional information about molecular evolution and increasing macrolide resistance of *Bordetella pertussis*, Shanghai, China, 2016–2022.
