# Supplementary material for: Molecular Evolution and Increasing Macrolide Resistance of Bordetella pertussis, Shanghai, China, 2016–2022
Source: Emerg Infect Dis. 2024 Jan;30(1):29–38. doi: 10.3201/eid3001.221588 (PMC10756392; doi:10.3201/eid3001.221588)
Supplement: Appendix 2 — Additional information about molecular evolution and increasing macrolide resistance of Bordetella pertussis, Shanghai, China, 2016–2022. [file 22-1588-Techapp-s2.pdf]

Article DOI: https://doi.org/10.3201/eid3001.221588

EID cannot ensure accessibility for supplementary materials supplied by authors. Readers who have difficulty accessing supplementary content should contact the authors for assistance.

Molecular Evolution and Increasing Macrolide Resistance of *Bordetella pertussis*, Shanghai, China, 2016–2022

Appendix 2

Appendix 2 Table 1. The age distributions of 1065 pertussis cases from 2016 to 2022 (n)

	Year						
Age groups	2016	2017	2018	2019	2020	2021	2022
0–1	11	157	174	160	9	38	82
>1–3y	0	13	7	19	0	3	31
>3–5y	0	4	1	4	0	16	49
>5–10y	0	3	2	6	0	75	183
>10y-12y	0	0	0	0	0	0	18
Total	11	177	184	189	9	132	363

Appendix 2 Table 2. The VNTRs profiles of fourteen MLVA types in this study

		MLVA profiles					
MLVA types	Number	VNTR1	VNTR3a	VNTR3b	VNTR4	VNTR5	VNTR6
MT195	76	8	6	0	7	6	8
MT28	74	8	7	0	7	6	8
MT27	59	8	7	0	7	6	7
MT104	38	8	6	0	7	6	10
MT55	18	8	6	0	7	6	9
MT158	3	8	7	0	7	7	7
MT16	3	8	6	0	7	6	7
MT29	2	8	7	0	7	6	9
MT114	2	8	7	0	7	6	4
MT30	1	8	7	0	7	6	10
MT32	1	8	7	0	8	6	7
untyped-1	1	8	7	0	8	6	14
untyped-2	4	8	6	7	7	6	5
untyped-3	1	8	6	0	7	5	3

Appendix 2 Table 3. Virulence genotype profiles and MLVA types of 283 BP isolates

Virulence genes allele and genotype profiles		MLVA types	Number	Frequency
ptxP	*ptxP1*	–	141	49.8%
	ptxP3	–	142	50.2%
fhaB	*fhaB1*	–	145	51.2%
	fhaB3	–	138	48.8%
prn	*prn1*	–	141	49.8%
	prn2	–	140	49.5%
	prn3	–	1	0.4%
	prn9	–	1	0.4%
ptxC	*ptxC1*	–	141	49.8%
	ptxC2	–	141	49.8%
	ptxC3	–	1	0.4%
ptxA	*ptxA1*	–	283	100.0%
	ptxA2	–	0	0.0%
fim2	*fim2–1*	–	283	100.0%
	fim2–2	–	0	0.0%
fim3	*fim3–1*	–	278	98.2%
	fim3–2	–	2	0.7%
	fim3–4	–	3	1.1%
ptxP1/prn1/fhaB3/ptxC1/ptxA1/fim2–1/fim3–1		16,27,30,55,104,195, untyped-3	138	48.7%
ptxP3/prn2/fhaB1/ptxC2/ptxA1/fim2–1/fim3–1		27,28,32,114,158, untyped-2	135	47.7%
ptxP3/prn2/fhaB1/ptxC2/ptxA1/fim2–1/fim3–2		27	3	1.1%
ptxP1/prn1/fhaB1/ptxC1/ptxA1/fim2–1/fim3–4		29, untyped-1	3	1.1%
ptxP3/prn2/fhaB1/ptxC1/ptxA1/fim2–1/fim3–1		28	2	0.7%
ptxP3/prn3/fhaB1/ptxC3/ptxA1/fim2–1/fim3–1		27	1	0.4%
ptxP3/prn9/fhaB1/ptxC2/ptxA1/fim2–1/fim3–1		28	1	0.4%
